# Complex organochlorine pesticide mixtures as determinant factor for breast cancer risk: a population-based case–control study in the Canary Islands (Spain)

**DOI:** 10.1186/1476-069X-11-28

**Published:** 2012-04-25

**Authors:** Luis D Boada, Manuel Zumbado, Luis Alberto Henríquez-Hernández, Maira Almeida-González, Eva E Álvarez-León, Lluis Serra-Majem, Octavio P Luzardo

**Affiliations:** 1 Toxicology Unit, Dpt. of Clinical Sciences, Universidad de Las Palmas de Gran Canaria and Instituto Canario de Investigación del Cáncer (ICIC), P.O. Box 550, Las Palmas de Gran Canaria, 35080, Spain; 2 Radiation Oncology Department, Hospital Universitario de Gran Canaria Dr Negrín. Canary Health Service, C/Barranco de La Ballena s/n, Las Palmas de Gran Canaria, 35010, Spain; 3 Preventive Medicine Service, Complejo Hospitalario Insular-Materno Infantil, Canary Health Service and Instituto Canario de Investigación del Cáncer (ICIC), Avda. Marítima del Sur s/n, Las Palmas de Gran Canaria, 35016, Spain; 4 Preventive Medicine Unit, Dpt. of Clinical Sciences, Universidad de Las Palmas de Gran Canaria and Instituto Canario de Investigación del Cáncer (ICIC), P.O. Box 550, Las Palmas de Gran Canaria, 35080, Spain

**Keywords:** Breast cancer risk, Organochlorine pesticides mixtures, Dichlorodiphenyldichloroethane (DDD)

## Abstract

**Background:**

All the relevant risk factors contributing to breast cancer etiology are not fully known. Exposure to organochlorine pesticides has been linked to an increased incidence of the disease, although not all data have been consistent. Most published studies evaluated the exposure to organochlorines individually, ignoring the potential effects exerted by the mixtures of chemicals.

**Methods:**

This population-based study was designed to evaluate the profile of mixtures of organochlorines detected in 103 healthy women and 121 women diagnosed with breast cancer from Gran Canaria Island, and the relation between the exposure to these compounds and breast cancer risk.

**Results:**

The most prevalent mixture of organochlorines among healthy women was the combination of lindane and endrin, and this mixture was not detected in any affected women. Breast cancer patients presented more frequently a combination of aldrin, dichlorodiphenyldichloroethylene (DDE) and dichlorodiphenyldichloroethane (DDD), and this mixture was not found in any healthy woman. After adjusting for covariables, the risk of breast cancer was moderately associated with DDD (OR = 1.008, confidence interval 95% 1.001-1.015, *p* = 0.024).

**Conclusions:**

This study indicates that healthy women show a very different profile of organochlorine pesticide mixtures than breast cancer patients, suggesting that organochlorine pesticide mixtures could play a relevant role in breast cancer risk.

## Background

**Figure 1:**
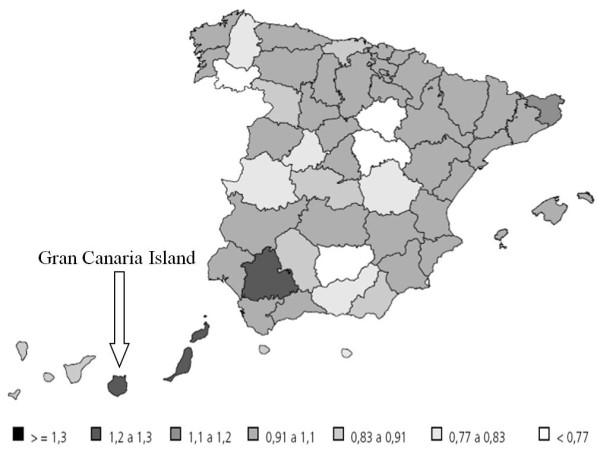
**Geographic distribution of mortality for breast cancer among women in Spain during the period between the years 1996 to 2000.** Numbers are referred to deaths per 100.000 inhabitants. (http://www.isciii.es/htdocs/centros/epidemiologia/libros/cancermsc.pdf).

Breast cancer (BC) is the most common cancer diagnosed in women worldwide [1]. In Spain, BC is currently the most significant cause of death from malignancies in women [2]. Whereas BC mortality and incidence are lower in mainland Spain than in other European countries, there are alarming rates of mortality because of this type of cancer in the archipelago of the Canary Islands, specifically in Gran Canaria Island (Figure 1) [3,4].

The etiology of BC is complex, with genetic, epigenetic and environmental factors contributing to the development of the disease. BC risk is significantly influenced by genetics, but over 70% of the women that are diagnosed have sporadic cancer or tumours not associated with inheritance of any major identified high risk genes. It is thought that the risk of BC can be modified by lifestyle and environment. Besides the genetic influence, the most established factors contributing to BC are related to cumulative exposure of the breast tissue to endogenous estrogens [5]. Thus, early menarche, late age at first pregnancy, nulliparity, lactation, years of reproductive life, hormonal contraception and hormone replacement therapy have been associated to an estrogenic environment and implicated as BC risk factors. Because all the relevant risk factors contributing to breast cancer etiology are not fully known, other environmental factors have been studied during the past decade, including smoking habits [6], alcohol intake [7], obesity, diet [8,9], and exposure to environmental contaminants [10,11]. Specifically, exposure to organohalogenated contaminants has been linked to BC etiology. Among environmental contaminants linked to BC, most studies have focused in organochlorine pesticides (OCPs) due to their endocrine-disrupting properties (estrogenic or antiandrogenic effects) observed both in *in vivo* and *in vitro* studies [12,13], and to their biochemical characteristics (high lipophilic and resistant to biotransformation) that result in their accumulation in body fat. In fact, their persistence in the environment leads to bioaccumulation in animals and also to biomagnification in the food chain [14], resulting in the bioaccumulation of these chemicals in the human body, including adipose tissue, fatty tissue, breast milk, or serum [15-18]. Their long half-lives in human tissues make them a problem for up to several decades [19-21]. Due to such circumstances, OCPs were banned in the 1970s in most Western countries [22,23]. A number of OCPs such as dichlorodiphenyltrichloroethane (DDT), aldrin or dieldrin, as well as its metabolites, are considered as xenoestrogens [16,24] and have been linked to environment-induced breast cancer [16,18,20]. Because dichlorodiphenyldichloroethylene (DDE, the major metabolite of DDT) is the most prevalent organochlorine residue found in human tissues [16,18,25], most studies have focused in the potential role played by DDE as a risk factor for BC. However, epidemiologic studies linking organochlorine pesticides (mainly DDT-derivative pesticides) to BC risk have been controversial. Although several earlier studies suggested a positive association [20,26-29], other studies showed no increased risk [30-32]. In any case, it has to be highlighted that most studies focused in the study of only one compound, and they did not take into account that human beings are exposed simultaneously to multiple OCPs, and that the biological effects exerted by the mixture of OCPs vary considerably from those exerted by any OCP individually [33,34].

The level of contamination by OCPs showed by the general population of the Canary Islands has been extensively studied. Despite the fact that most OCPs pesticides were banned in Spain in the late 1970’s, our results have already shown that the people living in the Canary Islands presented a relatively high degree of contamination by OCPs (including DDT and its derivatives, aldrin dieldrin, endrin, and lindane). Furthermore, such results seemed to indicate the existence of chronic exposure to OCPs that persisted in the late 1990’s [23,35].

Bearing in mind the alarming rates of mortality by BC among women from the Canary Islands (Spain) and more specifically in women from Gran Canaria Island [2,4], the aim of our study was to compare the profile of mixtures of OCPs detected in women diagnosed with BC living in Gran Canaria Island with that detected in healthy women selected among participants from our previous population-based studies, and secondarily to evaluate whether OCPs´ exposure could be considered as a risk factor for BC in such population.

## Methods

### Study group

Adult healthy women from Gran Canaria Island (n = 103) were selected among the representative population-sample obtained in the Canary Islands Nutrition Survey (ENCA 1998) previously evaluated regarding their OCPs serum levels. The characteristics and methodology of this Nutritional Survey and all the data about the level of contamination by OCPs showed by the population of this Archipelago have been previously published [23,35,36].

On the other hand, women with a histological confirmed first diagnosis of breast cancer (n = 121) were recruited between April 1999 and June 2001 from the two University Hospitals of Gran Canaria Island [37]. Cases and controls were selected according to place of residence (Gran Canaria Island). The characteristics of the studied population are shown in Table 1. The local ethics committee approved the design of this study, and informed consent was obtained from all the participants.

**Table 1:** Characteristics of the study population

	Healthy women (n = 103)	Patients (n = 121)	*P*
Age (years) Mean ± SD	45.3 ± 13.8	58.0 ± 11.7	<0.001
< 35	31 (30.1%)	2 (1.7%)	<0.001
35-44.9	23 (22.3%)	14 (11.6%)	
45-54.9	23 (22.3%)	35 (28.9%)	
55-64.9	17 (16.6%)	34 (28.1%)	
>65	9 (8.7%)	36 (29.7%)	
BMI (kg/m^2^) Mean ± SD	26.3 ± 4.3	27.7 ± 4.8	0.031
Lactation			
≤ 8 months	64 (84.2%)	63 (76.8%)	n. s.
>9 months	12 (15.8%)	19 (23.2%)	
Smoking			
Non-smokers	70 (70.7%)	95 (79.8%)	n. s.
Smokers/Ex­smoker	29 (29.3%)	24 (20.2%)	
Menopause			
Yes	6 (5.8%)	22 (18.2%)	0.024
No	77 (74.8%)	98 (81%)	
ND	20 (19.4)	1 (0.8%)	

*Abbreviations:* BMI, body mass index; SD, standard deviation; ND, not determined;

n. s., non-significant.

### Sample collection

Approximately 40 ml of blood was collected from each individual by venipuncture in a vacuum system tube, refrigerated, and centrifuged at 4°C (15 min x 3,000 rpm) within 4 hours to obtain serum. Serum was distributed in aliquots of 2–3 ml and immediately frozen at −80°C. The serum aliquots were used to determine biochemical (including lipid profile) and nutritional parameters. Once these initial analyses were completed, the remaining serum was kept frozen for further chemical analyses. All collection and handling equipment in contact with serum specimens were tested for possible OCP contamination. No contaminating material was identified.

### Analytical methods

The OCPs residues measured in serum were: DDT isomers 1,1,1-trichloro-2,2-bis(p-chlorophenyl)ethane (*p,p´*-DDT), 1,1,1-trichloro-2-(o-chlorophenyl)-2-(p-chlorophenyl)ethane (*o,p´*-DDT); DDT metabolites 1,1-dichloro-2,2-bis(p-chlorophenyl)ethylene (*p,p´*-DDE), 1,1-dichloro-2-(o-chlorophenyl)-2-(p-chlorophenyl)ethylene (*o,p´*-DDE), 1,1-dichloro-2,2-bis(p-chlorophenyl)ethane (*p,p´*-DDD), and 1,1-dichloro-2,(o-chlorophenyl)-2-(p-chlorophenyl)ethane (*o,p´*-DDD); the cyclodienes aldrin, dieldrin, and endrin; and the γ-isomer of hexachlorocyclohexane (lindane).

Chemical analysis procedure has been previously reported [23,35]. Briefly, quantitative analyses of the OCPs were undertaken on a gas-chromatography (GC)-electron capture detector (^63^Ni) (HP6890 with HP2397A μECD, Agilent Technologies Inc., CA, USA). A 5% phenyl methyl siloxane capillary column of 30 m x 0.22 mm i.d. x 0.25 μm (HP5 Supelco Co., Sigma-Aldrich Co., PA, USA) was used throughout the analyses. The GC operating conditions were as follows: carrier gas helium and argon-methane (95 + 5) as make-up gas at a flow rate of 1.5 ml/minute; pulsed splitless injection; an initial temperature of 50°C was held for 2 minutes, then the temperature was increased to 200°C at a rate of 30°C/minute, and finally to 280°C at a rate of 5°C/minute. The analytical limit of detection was 1 ppb (ng/g fat) for all the analytes. The accuracy of the analytical procedure was checked by using reference solutions of pure analytical grade.

The concentration of serum OCPs obtained in the chromatographic analysis was lipid-adjusted because lipid-adjusted serum concentrations of lipophilic compounds give a better estimation of the burden [38]. Total cholesterol and triglycerides were measured with an automatic Hitachi Analyzer 717 (Boehringer Manheim, IN, USA). Estimation of total serum lipids were calculated as previously reported [39,40]:

TL (total lipids) = 2.27 TC (total cholesterol) + TG (triglycerides) + 62.3

Due to the fact that *o,p’-*DDT, *o,p’-*DDE, and *o,p’-*DDD were detected in a small percentage of samples, they were not considered throughout this work. Thus, in this study we refer to *p,p’*- isomers of DDT, DDE and DDD as DDT, DDE and DDD, respectively. Similarly, we express the total DDT body burden as the sum of the three OCP-DDTs isomers more frequently measured (*p,p´*-DDT, *p,p´*-DDE, and *p,p´*-DDD); the total cyclodienes body burden (total Cyclodienes) as the sum of the three cyclodienes pesticides measured (aldrin, dieldrin and endrin); and the total OCPs body burden as the sum of the most frequently detected OCPs and metabolites measured (*p,p’-*DDT, *p,p’-*DDE, *p,p’-*DDD, aldrin, dieldrin, endrin, and lindane).

## Statistical analysis

In our sample, the distribution of OCPs deviates significantly from normality. For this reason, non-parametric tests were employed. Thus, Mann–Whitney *U*-test was used to explore differences in OCPs levels between groups. Chi square test was used to compare differences in categorical variables. Correlation between OCP levels and continuous variables was analyzed with the Spearman’s correlation test. In order to explore the possibility that other factors could be determinants to OCPs serum levels, multivariate analysis (binary logistic regression) test was used, adjusting for the well-known confounding variables, such as age, body mass index (BMI), menopausal status, lactation and smoking habits. All test were two sided and considered as significant if P < 0.05.

## Results

The studied population included 103 healthy women and 121 women diagnosed of BC, all of them from Gran Canaria Island. Healthy women were younger than BC patients (*p* < 0.001). Additionally, BMI values were higher in BC patients than in healthy women (*p* = 0.031). Related to menopausal status, most women from both groups were premenopausal women (74.8 and 81% in healthy and affected women, respectively). We observed statistical differences regarding to the distribution of menopausal status in the population (*p* = 0.024). We did not observe statistical differences between groups regarding to other demographical variables (Table 1).

A clear correlation between age and OCPs serum levels was evident in the group of healthy women. Thus, a positive relation between age and *p,p´*-DDE serum values was evident (*r* = 0.320; *p* = 0.001), while serum values of aldrin (*r* = −0.345; *p* < 0.001) and lindane (*r* = −0.242; *p* = 0.014) were negatively related to age. On the contrary, we did not observe these associations in the group of women affected of BC.

All analyzed samples had detectable residues of any OCP; samples from BC patients had a higher number of residues (an average of 3.66 different OCP residues per sample; range: 1–6; median: 4) than samples from healthy women (mean 3.25 OCPs per sample; range 2–7; median 3) (*p* = 0.008).

**Figure 2:**
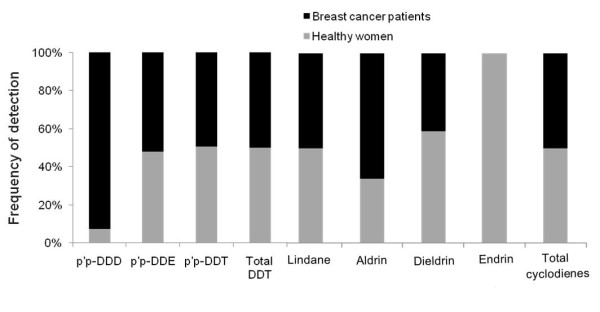
Frequency of detection of organochlorine pesticides among breast cancer patients and healthy women.

As shown in Table 2, DDE (the main DDT-metabolite) was detected in most samples from healthy women and BC patients (89 and 97%, respectively). The other DDT-metabolite, DDD, was also found in a high number of serum samples (72%) from women suffering BC, but it was detected in a small number of healthy subjects (6%) (*p* < 0.001). Interestingly, the main component of technical DDT (*p,p´*-DDT), used in commercial formulations of DDT and banned in Spain in last 1970’s, was present in more than 70% of the samples from both groups of women. With respect to non-DDT-derivatives, we observed discrepant results. Thus, despite the fact that the presence of residues of lindane and dieldrin was similar in both groups of women (more than 20% in both cases), the percentage of samples showing detectable levels of aldrin was clearly higher in BC patients than in healthy women (74 *vs.* 38%; *p* < 0.001). On the contrary, samples from healthy women showed a high presence of residues of endrin compared with samples from affected women (58 *vs.* 0%, respectively) (Table 2). Interestingly, these differences were observed also in the subgroup of subjects older than 45 years old (Table 3). The relative prevalence of OCPs in both groups of women is summarized in Figure 2.

**Table 2:** Levels of organochloride residues (ng/g lipid) detected in serum samples in healthy women and in women diagnosed from breast cancer

	Healthy women (n =103)			Breast cancer women (n =121)			
	Mean ± SD	Median (p5-p95)	D (%)	Mean ± SD	Median (p5-p95)	D (%)	*P*
Lindane	24.7 ± 43.0	0.0 (0.0-111.4)	28 (27.2%)	53.2 ± 88.8	0.0 (0.0-220.0)	33 (27.3%)	n.s.^a^ n.s.^b^
Aldrin	27.1 ± 37.9	0.0 (0.0-100.1)	39 (37.9%)	72.5 ± 107.2	75.8 (0.0-116.4)	90 (74.4%)	<0.001^a^ <0.001^b^
Dieldrin	9.5 ± 16.4	0.0 (0.0-46.2)	33 (32.0%)	12.6 ± 29.5	0.0 (0.0-72.0)	27 (22.3%)	n.s.^a^ n.s.^b^
Endrin	231.4 ± 444.4	29.1 (0.0-1279.0)	60 (58.3%)	-	-	0 (0%)	n.a.^a^ n.a.^b^
Total Cyclodienes	268.1 ± 443.3	91.4 (0.0-1297.8)	81 (78.6%)	85.2 ± 117.7	79.9 (0.0-156.1)	95 (78.5%)	n.s^a^ 0.027^b^
p,p-DDE	198.0 ± 207.5	167.7 (45.0-706.0)	92(89.3%)	357.4 ± 326.0	300.1 (106.1-653.3)	118 (97.5%)	0.012^a^ <0.001^b^
p,p-DDD	21.8 ± 131.2	0.0 (0.0-129.2)	6 (5.8%)	440.3 ± 412.7	551.1 (0.0-1108.2)	87 (71.9%)	<0.001^a^ <0.001^b^
p,p-DDT	319.5 ± 417.4	217.0 (0.0-1428.6)	77 (74.8%)	149.3 ± 111.7	153.0 (0.0-327.9)	88 (72.7%)	n.s.^a^ <0.001^b^
Total DDTs	880.8 ± 971.9	665.0 (69.1-3622.0)	103 (100%)	1049.6 ± 679.5	979.3 (159.2-2228.7)	121 (100%)	n.s.^a^ 0.001^b^
Total OCPs	1173.6 ±1339.9	706.0 (242.7-5263.6)	103 (100%)	1188.1 ± 657.2	1112.8 (184.2-2375.9)	121 (100%)	n.s.^a^ 0.001^b^

*Abbreviations:* SD, standard deviation; D, detected compound; p5-p95, percentiles 5 and 95 of the distribution; n.s., non-significant; n.a., not applicable.

^a^*χ*^2^ test.

^b^ Mann–Whitney *U*-test.

**Table 3:** Levels of organochloride residues (ng/g lipid) detected in serum samples in healthy women and in women diagnosed from breast cancer older than 45 years old

	Healthy women (n = 49)			Breast cancer women (n =105)			
	Mean ± SD	Median (p5-p95)	D (%)	Mean ± SD	Median (p5-p95)	D (%)	*P*
Lindane	16.6 ± 34.6	0.0 (0.0-104.5)	10 (20.4%)	49.5 ± 85.4	0.0 (0.0-215.2)	27 (25.7%)	n.s.^a^ n.s.^b^
Aldrin	15.0 ± 27.5	0.0 (0.0-73.2)	12 (24.5%)	71.7 ± 114.3	74.8 (0.0-115.7)	76 (72.4%)	<0.001 ^a^ <0.001 ^b^
Dieldrin	7.0 ± 16.0	0.0 (0.0-49.7)	10 (20.4%)	12.2 ± 30.0	0.0 (0.0-71.7)	22 (21.0%)	n.s.^a^ n.s.^b^
Endrin	234.2 ± 466.0	0.0 (0.0-1553.1)	24 (49.0%)	-	-	0 (0%)	n.a.^a^ n.a.^b^
Total Cyclodienes	256.1 ± 463.6	46.5 (0.0-1553.1)	33 (67.3%)	83.9 ± 125.2	77.5 (0.0-156.7)	80 (76.2%)	n.s.^a^ n.s.^b^
p,p-DDE	275.6 ± 266.2	220.8.7 (0.0-653.1)	46 (93.9%)	354.5 ± 327.3	303.8 (106.1-648.8)	102 (97.1%)	n.s.^a^ 0.003^b^
p,p-DDD	8.6 ± 38.3	0.0 (0.0-94.3)	3 (6.1%)	412.4 ± 412.1	126.5.1 (0.0-1094.7)	72 (68.6%)	<0.001^a^ <0.001^b^
p,p-DDT	324.8 ± 395.5	205.4 (0.0-1334.6)	40 (81.6%)	153.2 ± 114.0	158.7 (0.0-327.3)	76 (72.4%)	n.s.^a^ 0.008^b^
Total DDTs	937.3 ± 824.2	706.0 (133.2-3017.4)	49 (100%)	1022.7 ± 684.7	892.7 (143.0-2225.8)	105 (100%)	n.s.^a^ n.s.^b^
Total OCPs	1210.1 ± 1243.6	729.7 (267.9-4570.5)	49 (100%)	1156.0 ± 661.1	1057.3 (181.7-2364.8)	105 (100%)	n.s.^a^ 0.043^b^

*Abbreviations:* SD, standard deviation; D, detected compound; p5-p95, percentiles 5 and 95 of the distribution; n.s., non-significant; n.a., not applicable.

^a^*χ*^2^ test.

^b^ Mann–Whitney *U*-test.

As shown in Table 2 median value of DDT was higher among healthy women than in affected women (217 vs. 153 ng/g lipid, respectively; *p* < 0.001). However, regarding to DDE and DDD the residue levels were higher among BC patients (*p* < 0.001 for both cases). As a consequence, serum samples from affected women showed higher median values of total DDT body burden (around 980 ng/g fat) than those values found in healthy women (665 ng/g fat) (*p* = 0.001). On the contrary, total cyclodienes body burden was higher in healthy women than in serum samples from BC patients (*p* = 0.027).

Nevertheless, if we consider the total OCPs residues as the sum of the three DDT-derivatives measured and the sum of the three cyclodienes measured plus lindane, we could observe that median levels of total OCP compounds were higher in BC subjects than in healthy women (*p* = 0.001) (Table 2). Moreover, this difference was also observed in the subgroup of healthy women and BC patients older than 45 years old (*p* = 0.043) (Table 3).

As stated previously, among BC patients, 30 subjects (24.8%) presented a combination of aldrin, DDE, and DDD, followed by DDD alone and DDD and DDE (10 subjects). Interestingly, we did not observe overlaps between these OCP-mixtures among both groups of women, that is: no healthy subject had combination of residues of aldrin plus DDE plus DDD, and no BC patient had combination of residues of lindane plus endrin.

Finally, since age, BMI and menopausal status seem to play an important role in BC etiology [41], and having into account that these parameters and others (such as lactation, and smoking habits) seem to be important determinants of the levels of contamination by OCPs [23,35,42], we carried out a multivariate analysis, including all the pesticides simultaneously in the model and adjusting by age, BMI, menopause status, lactation and smoking habits; in order to evaluate the existence of any association between OCPs´ exposure and a BC risk. As shown in Table 4, among the OCPs evaluated, only *p,p’*-DDD showed a moderate increment in the risk of developing BC (odds ratio 1.008 [95%CI, 1.001-1.015], p = 0.024).

**Table 4:** Binary logistic regression of pesticide in multivariate analysis adjusted by age, BMI, menopausal status, lactation and smoking habits

Pesticide	Odds ratio, 95% CI	*P*
Healthy women	(Ref. category)	
Lindane	1.097 (0.420-28.412)	0.988
Aldrin	1.027 (0.991-1.065)	0.147
Dieldrin	1.002 (0.956-1.050)	0.927
Endrin	N.A.	
*p,p’*-DDD	1.008 (1.001-1.015)	0.024
*p,p’*-DDE	0.999 (0.996-1.001)	0.284
*p,p’*-DDT	0.994 (0.987-1.002)	0.139

*Abbreviations:* 95% CI, 95% confidence interval; N.A., not applicable.

## Discussion

Although the vast majority of epidemiologic studies do not support the association between BC and OCPs [15,20,32,43,44], the well-known association between BC and prolonged exposure to estrogens suggests that environmental estrogens, may play a critical role in the cellular and molecular changes that occur during breast carcinogenesis [45]. In this sense, most OCPs are considered as xenoestrogens and may modulate steroid sex hormones homeostasis, such as estrogen or testosterone, as agonists or antagonists or as mixed effects [46]. In fact, there are a number of studies that seem to indicate that the association between BC and OCPs could exist [27,47].

The best known and studied OCP with respect to its role in breast cancer is the main DDT-metabolite, DDE. DDT is converted to DDE, that apparently does not undergo further biotransformation, and it is stored for an indefinite period of time in adipose tissues. Due to this fact, this compound is the highest prevalent DDT-derivative found in human beings [18]. On the other hand, the major detoxification pathway of DDT is via dechlorination to DDD. The population of the Canary Islands presents similar serum levels of DDT and DDE to those found in other European countries [23]. In the present study, serum levels of DDE were higher in BC patients than in healthy women, although, after a multivariate analysis, DDE do not seem to be a risk factor for BC. These results agree with those reported previously [32]. However, there are other highly prevalent OCPs (DDT- and non-DDT-derivatives) in female populations at concentrations highly enough to exert biological effects on breast cells [34]. In this sense, our findings demonstrating that serum levels of DDD were higher among BC patients than in non-affected women and that exposure to DDD could represent, at least, a moderately risk factor for developing BC (OR = 1.008), point to the possibility that other less evaluated OCPs, such as DDD, could play a more relevant role as risk factor for BC.

We reported that background exposure to non-DDT-derivative-OCPs was higher in non-affected women than in BC patients. This result was mainly due to the fact that endrin was not detected in any sample from BC patients, while residues of endrin were present in more than 58% of the samples from healthy women at median concentrations higher than 29 ng/g fat. On the contrary, the presence of residues of aldrin was higher in serum samples from BC patients than in healthy women. Thus, depending on the specific subclass of OCP, the environmental exposure to these contaminants might be related or not with the etiology of BC. Our results showing that DDD could be considered as risk factors for BC must be taken with caution due to the several limitations of our study. Firstly, controls were originally selected for a different purpose (ENCA survey) than analyzed in this study. The use of controls from a nutritional survey may introduce bias if the probability of selection into the study is associated with factors that influence both the prevalence of exposure and the probability of disease status. One such factor is age, which was statistically different among groups of subjects (47.6% of healthy women were older than 45 years old while 86.8% of BC patients were in this age subgroup); together with BMI or menopause status (both variables also considered as BC risk factors and important for the prevalence of exposure to OCPs). Secondly, several established risk factors for breast cancer (mainly family history of breast cancer, reproductive factors (such as number of children or duration of lactation), or alcohol intake) were not available nor in controls nor in BC patients, and, as a consequence, unmeasured or residual confounding factors have not been included in the analyses. Thirdly, because these chemicals are stored in fat, it is believed that adipose tissue loss could result in increased \organ and blood concentrations of these compounds [48]. Thus, if changes in body weight occur as a consequence of the development of cancers, the levels of OCPs measured in this study may not reflect the background exposure to these chemicals for both groups of women. In addition, body fat is an important risk factor for breast cancer [49]. Although we adjusted for BMI in our analyses, BMI as a measure of body fat has certain limitations [50]. To make matters worse, because serum concentrations of OCPs are highly correlated with each other and can be correlated with unmeasured substances, the associations observed here might not always have represented the direct effect of the OCPs measured.

It is assumed that the biological effects exerted by environmental contaminants on human tissues, taken individually, clearly differ from the effects exerted by their combinations. However, most studies about environmental contaminants as risk factors for BC have focused on single-chemicals. Currently, it seems clear the importance of the exposure to chemical mixtures and their contribution to the disease by causing cellular-level dysfunction along key pathways. In this sense, we have recently reported that DDD, DDE, aldrin, and dieldrin, sharply upregulated the expression of a number of protein kinases genes that could be involved in the etiology of BC, such as ACVRL1, ALK-1, KIT, ERBB3, and ALK-1, at concentrations close to those detected in human populations [34]. Additionally, it has to be taken into account that the potential effects exerted on breast cells by environmental contaminants could be due not only to the organochlorine pesticides measured in the present study, even more so other environmental pollutants not measured could be implicated. Complex interactions between chemicals, endogenous or exogenous hormones, and their natural ligands and receptors may alter the internal homeostasis of the estrogenic environment of mammary tissue, leading to malignant transformation and cancer. Our findings agree with those observed in vitro by Aubé (2011) suggesting that aldrin and DDD, together with other OCP compounds, increased the proliferation of MCF-7 cells [51]. However, to our knowledge, population-based studies evaluating the role of OCPs mixtures in relation to breast cancer are scarce [52]. Our findings showing the lack of overlaps between the profiles of mixtures of OCPs among healthy and affected women suggest a relevant role of these chemical mixtures in relation to the disease. Future studies achieving the association between environmental contaminants and BC should analyze the combined effect of these compounds and the interactions with endogenous hormones and other substances that affect endocrine function.

## Conclusions

In conclusion, our results indicate that serum concentrations of OCPs were higher in BC patients than in healthy women in this specific sample of the Spanish population, and suggest that the combination of certain OCPs could be highly relevant in the potential role played by these chemicals as risk factors for BC. More specifically, our results seem to indicate that the mixture of aldrin plus DDE plus DDD, may play a relevant role of as a potential environmental factor related to BC. In this context, despite the controversial and complex issues involved with continued exposure to residues of OCPs through the environment and food, their potential role as risk factor for BC, should be considered by Public Health Authorities in countries where BC alarming rates of BC incidence or mortality exists.

## Abbreviations

BC, Breast cancer; BMI, Body mass index; DDT, Dichlorodiphenyltrichloroethane; DDE, Dichlorodiphenyldichloroethylene; DDD, Dichlorodiphenyldichloroethane; OCPs, Organochlorine pesticides.

## Conflict of Interest

The authors declare that there are no conflicts of interest.

## Authors’ contribution

LDB has made substantial contributions to conception and design, has participate in analysis and interpretation of data, has been involved in drafting the manuscript and has given final approval of the version to be published; MZ has made substantial contributions to conception and design and has been involved in acquisition of data; LAHH has participate in analysis and interpretation of data and has been involved in drafting the manuscript; MAG has been involved in acquisition of data; EEAL has participate in analysis and interpretation of data; LSM has revised the manuscript critically for important intellectual content; OPL has made substantial contributions to conception and design and has been involved in acquisition of data and has revised the manuscript critically for important intellectual content. All authors read and approved the final manuscript.
